# Training in communication skills for self-efficacy of health professionals: a systematic review

**DOI:** 10.1186/s12960-021-00574-3

**Published:** 2021-03-06

**Authors:** Ádala Nayana de Sousa Mata, Kesley Pablo Morais de Azevedo, Liliane Pereira Braga, Gidyenne Christine Bandeira Silva de Medeiros, Victor Hugo de Oliveira Segundo, Isaac Newton Machado Bezerra, Isac Davidson Santiago Fernandes Pimenta, Ismael Martinez Nicolás, Grasiela Piuvezam

**Affiliations:** 1 Multicampi School of Medical Sciences of Rio Grande do Norte, Federal University of Rio Grande do Norte, Av. Coronel Martiniano, 541, Centro, Caicó, RN ZIP Code: 59300-000 Brazil; 2 Post-Graduate Program in Public Health, Health Science Center, Federal University of Rio Grande do Norte, Av. Sen. Salgado Filho, 1787 - Lagoa Nova, Natal, RN ZIP Code: 59056-000 Brazil; 3 Department of Nutricion, Federal University of Rio Grande do Norte, University Campus, Av. Senador Salgado Filho, 3000, Lagoa Nova, Natal, RN ZIP Code: 59.078-970 Brazil; 4 Academic Center of Vitória, Federal University of Pernanmbuco, R. Aldo do reservatório, s/n, Bela Vista, Vitória de Santo Antão, PE ZIP Code: 55608-680 Brazil; 5 Department of Health Sciences, Catholic University San Antonio de Murcia, San Antonio de Murcia, Campus de los Jerónimos, 135, 30107 Guadalupe, Murcia Spain; 6 Department of Public Health, Federal University of Rio Grande do Norte, University Campus, Av. Senador Salgado Filho, 3000, Lagoa Nova, Natal, RN ZIP Code: 59.078-970 Brazil

**Keywords:** Communication, Health personnel, Self-efficacy, Training, Systematic review

## Abstract

**Background:**

Communication skills are essential for health professionals to establish a positive relationship with their patients, improving their health and quality of life. In this perspective, communication skills training can be effective strategies to improve the care provided by professionals in patient care and the quality of health services.

**Objective:**

To identify the best available evidence on training programs in communication skills to promote changes in attitude and behavior or self-efficacy of health professionals.

**Methods:**

Systematic searches were performed in eight databases, evaluating Randomized Controlled Trials and quasi-experimental studies with a control group, focusing on training communication skills for health professionals, who assessed self-efficacy or behaviors related to these skills. The phases of study selection and data extraction were carried out by two independent researchers, and the conflicts were resolved by a third. The risk of bias was assessed using the Cochrane method.

**Results:**

Eight studies were included in the review. Most programs lasted between 4½ h and 2 days, involved information about communication skills and the content was applied to the health professionals’ context. Several teaching strategies were used, such as lectures, videos and dramatizations and the evaluation was carried out using different instruments. Improvements in the performance and in the self-efficacy of communication skills were observed in the trained groups. The RCT had a low risk of bias and the quasi-experimental studies had a moderate risk.

**Conclusion:**

Training in communication skills can improve the performance and self-efficacy of health professionals. Programs that approach the conceptual issues and promote the space for experiential learning could be effective in communication skills training for professionals.

*PROSPERO*: CRD42019129384

**Supplementary Information:**

The online version contains supplementary material available at 10.1186/s12960-021-00574-3.

## Background

Communication skills (CS) consist of the efficient transmission of information, including verbal communication, such as speech units and listening strategies, and non-verbal communication, such as gestures and expressions, eye contact and body language. They are an instrument that can enable the understanding and processing of information by the patient, during the care of health professionals, through empathy, informed collaborative choice and patient involvement [1, 2]. CS, when centered on the patient, leads health professionals to identify demands and plan treatment through knowledge and the provision of a therapeutic and supportive environment for shared decision-making [3], and enables greater adherence to treatment and changes in behavior [4].

The encounter between professionals and patients involves different aspects, such as the patient's needs, the suffering he manifests, and the emotional availability of the professional, which do not allow the predictability of the lived experiences, thus justifying the need for communication about health care aspects to be a priority [5]. Attree [6] points out that patients understand communication with professionals as being necessary for quality care, and consider communication useful when it is carried out in a constructive, encouraging and supportive way. Patients' complaints focus on the perceived communication failure and the inability to adequately convey a sense of care [7]. Thus, the emotional aspects that involve communication with the patient are presented as a challenge for professionals and health services [8].

Training on how to inform patients about their health condition, illnesses and treatment; establishing relationships based on empathy, support and comfort; and promoting personal reflection on their communicative actions and interdisciplinary collaboration are essential for health professionals [9]. Communication skills training (CST) can have a beneficial effect on the self-efficacy of professionals [10], on improving services, and on the possibility of minimizing errors, which should be a priority, considering that these skills cannot be improved with just clinical experience [11].

The development of CST has been carried out in different health contexts (primary and tertiary care), and usually involves behaviors with high emotional burdens, such as delivering bad news [12]. The acquisition of new communication skills can improve the relationship with patients, especially when the training process occurs in an experiential way. In this context, when promoting the teaching of communication skills, cognitive, affective and behavioral components are incorporated, with the general objective of promoting greater self-awareness in health professionals [7].

One way to measure the impact of training is through self-efficacy, which is the individual belief about the ability to perform an activity with success. This construct states that individuals can modify their behavior based on the cognitive aspects and the relationships that they establish with their external environment. The perception of their skills and competences promotes a critical assessment of changes in performance and behavior, which can be favored by intervention and quality improvement programs [13]. Self-efficacy has been a widely used construct for self-assessment of the result of communication skills, as it is believed to have a direct influence on personal performance in specific contexts, considering the changes that can occur in behavior [14].

In a study by Kissane et al. [15] it was observed that the CST shows inconsistency between studies, both related to the concept of communication skills, and to the exposed content, program design, intervention time and results. This multiplicity of strategies and the performance of evaluations without methodological rigor and comparability make it difficult to identify an ideal program with an adequate structure and methods for teaching communication skills [16, 17]. Therefore, the objective of this systematic review was to identify the best evidence available, considering the greater methodological robustness, on the available training programs in communication skills, addressing aspects of structure, content, teaching and assessment strategies, and to present an effective model for promoting changes in attitude and behavior or self-efficacy in health professionals.

## Methods

### Search strategy

This systematic review study was registered in PROSPERO (CRD42019129384) (see Additional file 1), considering the Preferred Reporting Items for Systematic Reviews and MetaAnalyses [18] statement guidelines. The methodology is detailed in Mata et al. [19].

A broad search was carried out until April 2020, in the following databases: PubMed/Medline, Scopus, Web of Science, EMBASE, Science Direct, CINAHL, PsycINFO and the Cochrane Central Register of Controlled Trials (CENTRAL). The search strategy used Medical Subject Headings (MeSH) (see Additional file 2: Chart 1), and the manual search was carried out by investigating the references of the included articles.

### Selection of studies

This review selected Randomized Controlled Trials (RCT) and quasi-experimental studies with a control group, as the most appropriate methodological strategy for assessing the usefulness of the intervention.

Studies were included in which the intervention performed was the training of communication skills with health professionals, who reported changes in self-efficacy, or changes in attitude and behavior, related to communication skills and evaluated by the professionals themselves, without language restrictions.

Exclusion criteria were: studies carried out with undergraduate or graduate students, evaluations performed by patients, interventions carried out by mindfulness programs, psychotherapy or Balint group. Studies with incomplete descriptions of the intervention or results were excluded, if we cannot access the information through the contact with the authors of the research.

Two reviewers screened all abstracts and full-texts articles independently and blindly using Rayyan, a web and mobile app for systematic reviews [20]. Disagreements between the reviewers were resolved by discussions or with the help of a third reviewer.

### Data extraction and evaluation of methodological quality of studies

The data were extracted by two reviewers (INB and IDSFP). A file with the data extraction tables was sent to each review, who worked independently and blindly. The data extraction tables was tested and refined in a pilot study. A third reviewer (AM) resolved the divergences and organized the extracted data. Data were extracted to characterize the studies (sample and method), intervention data (period, content and teaching strategies) and evaluation (outcome, instruments and main results).

The risk of bias was assessed for all of the included articles following the recommendations of the Cochrane Handbook for Systematic Review of Interventions [21]. For the evaluation of the RCT tests, the bias risk tool (RoB) was used, which has structured domains to assess the risk of bias in the process of randomization, blinding, outcomes and overall bias. The risk of bias was assessed as low, high, or some concerns [22]. The software Review Manager 5.3 was used to elaborate the figures that summarize the evaluation of the risk of bias in clinical trials.

Non-RCTs were evaluated by the “Risk of Bias in Non-Randomized Studies of Interventions” tool (ROBINS-I) [23], which domains address risk assessment of bias before, during and after intervention. The domains can be classifieds as: (1) low-risk of bias; (2) moderate-risk of bias; (3) serious-risk of bias; (4) critical-risk of bias; and (5) no information.

Three reviewers (AM and INB; AM and IDSFP) independently assessed the methodological quality of the studies and any divergences were resolved by discussion or with the help of a fourth reviewer (GP). The quality of evidence was evaluated by Grading of Recommendations Assessment, Development and Evaluation (GRADE) guidelines, classified as very low, low, moderate and high [24]. The articles were not excluded based on the quality assessment.

## Results

The systematic search resulted in 2255 articles, and 379 references were identified through manual bibliographic review. A total of 8 articles met the criteria for eligibility in the study, according to PRISMA Flowchart—Fig. 1 (see Additional file 3).

The eight studies included in the systematic review were developed with target group of doctors and/or nurses, with a predominance of women, and three were conducted in the context of Primary Health Care. The sample size of the studies was reduced, varying between 15 and 35 health professionals, excepted for the study by Liu et al. [25] conducted with 117 nurses. In all studies, the control groups were not subjected to any intervention or activity. The details of the characteristics of the studies are shown in Table 1 (see Additional file 4).

### Characteristics of intervention programs

The results about the structure, content, teaching strategies and evaluation of the training programs in communication skills are presented in Table 2 (see Additional file 5). An important component of the intervention refers to the duration of the programs, which can be short (< 20 h) or long (≥ 20 h) [26]. Thus, the programs included in this review are considered, for the most part, to be of short duration with variation between 4½ h and 2 days. As an exception, the study by Ammentorp et al. [27] lasted for 5 days (34 h), and monitored groups, with an assessment at 3 and 6 months after the intervention. Two studies took breaks between training sessions to develop educational strategies, such as recording videos [27, 28].

The study developed by Levinson and Roter [29] evaluated the effect of two types of intervention, with different structures, which were submitted to different methodological designs. One, of short duration (4½ h), was characterized as a RCT, while the other, described by the authors as long-lasting (2½ days), and without a control group, was developed using a quasi-experimental study method. Thus, in this study, data related to the short-term program were considered, according to the inclusion criteria of the studies in this review.

### Content of interventions

The programs begin training covering the basic concepts of communication: communication models [30], fundamental interview skills [29, 31], interpersonal communication [32], spoken and written communication [33] and facilitating listening, non-verbal communication and assertive communication [28]. Liu et al. [25] identified the communication tasks that professionals most needed help with when are talking to patients, family members and colleagues. Specific communication skills were also addressed by Roter et al. [32]. Ammentorp et al. [27] explained the structure of the consultation before dealing with communication techniques, according to the model described by Maquire et al. [34] adopted in the intervention proposal.

Subsequently, some studies bring in their CST-specific themes to the context and the audience to which they are directed. In the pediatric context, strategies were developed to deal with the instrumental and affective needs of parents and children [31]. Sany et al. [33] directed the content to collaboration and counseling skills that promote patient self-care and self-efficacy, with a focus on understanding the patient and adhering to the treatment of arterial hypertension. In the perspective of training with oncology professionals, Fujimori et al. [30] addressed strategies for communicating bad news. Doyle et al. [28], aiming at providing training to deal with difficult communication situations, dealt with rescheduling and conflict resolution.

The studies by Ammentorp et al. [27] and Levinson and Roter [29], aimed at a general improvement in communication skills, inserted aspects focused on patient-centered attention. The first brought patient-centered attention by inserting content that addressed the understanding of patient’s preferences and needs, while the second exposed empathic care and patient involvement in discussions about health care.

### Teaching strategies

The eight studies presented different teaching resources used as a strategy for teaching communication skills, with proposals aimed at active and contextualized learning. To address general aspects of communication, Sany et al. [33] used strategies such as posters and graphics, relating them to patient education content, which was an objective of their intervention. The use of lectures and didactic presentations was used in the studies by Sany et al. [33], Fujimori et al. [30], Doyle et al. [28], Liu et al. [25] and Levinson and Roter [29]. The latter also used a case-based discussion with focus on interview skills. Ammentorp et al. [27] and Liu et al. [25] identified the tasks that professionals most need help with.

Role play was used as a learning strategy, with some variations in its execution: exercises [31, 33], discussion between peers [30] and with the use of feedback in small groups [27, 28]. Doyle et al. [28] proposed that the participants share their experiences and build scenarios for execution in role play. Liu et al. [25] used management support methods, in small groups, which included positive feedback, implementing teaching rounds, building models and performing role play in their workplaces.

The video projection resources were used to transmit content [25, 33], as a strategy to trigger discussions [25, 30] and for modeling and demonstrating behaviors [27, 28, 31]. The recording of video consultations with real patients for feedback was also used in the program by Ammentorp et al. [27]. van Dulmen and Holl [31] included theoretical and practical homework and application of what was learned between sessions. Learning exercises, reading material and a pocket card with the basic steps of the training content were distributed to the professionals who participated in the study by Doyle et al. [28]. Manuals with training material and reference on skills were distributed to participants in the Liu et al. [25] and Roter et al. [32] studies.

### Program evaluation

The assessment methods used in the training programs were diverse. Specifically to evaluate the communication skills outcome, the Health Literacy Assessment Questions (HLAQs) [33] and the evaluation through recorded visits with real patients evaluated by the Roter Interactional Analysis System (RIAS) [29, 32] were used, as well as evaluation of verbal and non-verbal communication using the camera computer system [31]. The performance of professionals in communication skills was carried out through the Objective Structured Clinical Examination (OSCE), assessed by 18 items from the AFLS (Awareness, Feelings, Listen, Solve) [28], and through simulated consultations punctuated by the SHARE items [30].

Fujimori et al. [30] also assessed the professionals' confidence using two instruments: 32 SHARE items and 21 items established by Baile et al. [35]. Self-efficacy was addressed directly in the studies by Doyle et al. [28], Ammentorp et al. [27] and Liu et al. [25], who used the questionnaire developed by Parle et al. [36]. In addition, Doyle et al. [28] also evaluated this outcome using the difficulty extension scale, which forms the composite scale together with the confidence scale. Liu et al. [25] it also assessed basic communication skills, self-efficacy and self-perceived support using specific instruments for nurses.

### Main results

In assessing the communication skills of professionals, Sany et al. [33] found a significant variation from the baseline measure for follow-up between the control (CG) and intervention (IG) groups (CG: 0.48 ± 9.08; IG: 17.33 ± 12.9). Fujimori et al. [30] identified a difference in the mean of the SHARE scores of confidence in the communication skills between the groups (IG: ∆ = 22.5 ± 34.4; CG: ∆ = − 17.1 ± 26.1; F = 13.7) and in the communication of bad news between groups (IG: ∆ = 19.2 ± 19.6; CG: ∆ = − 2.4 ± 15.4; F = 11.2). With regard to performance evaluation, the analysis of videos pointed out a significant difference in the moments before and after the intervention and between the groups.

The performance assessed using the OSCE, in Doyle et al. [28], showed that there was no significant difference between groups. Both groups improved performance: the IG by 2.6% and the CG by 5.3%, but there was no statistically significant difference between the groups (F = 3.46; *p* = 0.073). The study by Levinson and Roter [29] did not show any evidence of the effect of short-term training on the skills of professionals.

Self-efficacy, assessed by Doyle et al. [28], showed the most confident intervention group after the intervention on the composite scale (IG: 87.0; CG: 71.7; F = 24.4), on the confidence scale (IG: 91.9; CG: 73.9; F = 25.4) and on the difficulty scale (IG: 2.6; GC: 4.0; F = 8.1). The results presented by Ammentorp et al. [27] pointed out an increase in IG scores from T1 (before the intervention) to T2 (after the intervention) in all of the questions evaluated, and the increase in the average self-efficacy score, for each question, varied from 8.5 to 37%. The comparison of the mean scores of T1 and T2 showed a significant increase (Diff = 1.25; *p* ˂ 0.001), corresponding to an increase of 19%. The CG showed unchanged scores at all times of measurement.

The study by Liu et al. [25] revealed a significant improvement in the pre (T1) and post (T3) training period in the four dimensions evaluated: basic communication skills (T1: 268.87 ± 29.90; T3: 287.69 ± 33.85); self-efficacy (T1: 1247.45 ± 244.10; T3: 1430.39 ± 125.68); outcome expectancy (T1: 123.24 ± 17.56; T3: 132.32 ± 17.82) and self-perceived support (T1: 44.16 ± 5.78; T3: 50.16 ± 5.14).

In assessing some aspects of communication, Roter et al. [32] showed that trained doctors used significantly more facilitations in their visits and more open-ended questions, and van Dulmen and Holl [31] trained pediatricians seemed to express more agreement, to provide more medical information, and a to do more psychosocial question after the measurement.

### Methodological quality

In the RCTs, details of the randomization process were not observed and information about the blinding of participants and professionals was also not described. Even so, the risk of bias analysis showed that the outcomes are reported in the studies, with low bias (see Additional file 6: Fig. 2). In the assessment of non-RCTs, one of the studies was rated moderate bias (see Additional file 7: Table 3). The quality of the evidence from the included studies, assessed by GRADE, was classified as moderate.

## Discussion

This systematic review retrieved eight studies that addressed training programs in communication skills for the health professional and patient relationship. Improvements were observed in the performance and self-efficacy of professionals with regard to communication skills, through different teaching strategies, involving experiential activities, which are fundamental for the improvement of care and for patient-centered attention.

Moore et al. [7], when addressing the training of professionals for the care of cancer patients, pointed out that interventions aimed at professionals present small or inconclusive results. Also, in the study developed by Selman et al. [37], in the context of palliative care, it was shown that the analysis of results of training in communication skills is hampered by the variety of approaches, as well as by the low methodological quality of the studies and the difficulty in convincing professionals to participate. Thus, this review sought to include more methodologically systematized studies, and was not limited in terms of addressing performance scenarios or health conditions, seeking to identify the most appropriate structures, contents, teaching and evaluation strategies to ensure effectiveness in improving the self-efficacy and performance of health professionals in a broad way.

CST should be investigated, as this is important for the development of empathic and communication skills, enabling reflection on professional development and its relationship in work teams [37, 38]. However, the analyzed studies are aimed at doctors and/or nurses, not considering other health professionals. Consider the need for a comprehensive and multidisciplinary perspective of patient care, it is necessary to involve other professionals in training, to improve teamwork, the culture of communication in clinical practice and centered patient care [39, 40].

The context in which these studies were carried out can be predictors of training needs. Studies carried out in Primary Health Care are important, since the context requires professionals to be closer and more connected to the patient, and training can improve communication and favor the strengthening of safe and high-quality care [41, 42]. The benefits for patients are also assessed, especially in the context of chronic health conditions, in which interventions for professionals favor the follow-up and adherence to treatment, the resolution of symptoms and the control of pain and physiological measures [43, 44].

Another relevant aspect to be considered in training is the duration of the intervention, since time has been proven to be a fundamental aspect to guarantee reflection on the communicative process; this is because participants have the opportunity to produce, interpret and respond to communicative acts [45]. In this sense, proposals lasting a few hours and without the use of role play, as carried out by Levinson and Roter [29], may not have effective results in changing the behavior of health professionals, since studies point out that long-term programs (≥ 20 h) are more effective [26].

The continuity of programs must be an element to be considered for good results in the long-term. The study by Ammentorp et al. [27] pointed out that communication skills remain after 6 months of the intervention, corroborating the study by Fallowfield et al. [46], who identified that doctors even integrated communication skills into their practice 15 months after training. In this context, services face the challenge of ensuring the development and continuity of programs, ensuring that new employees are trained, as well as having refresher courses held frequently [38].

With regard to the content taught in the training, the studies included initially clarify concepts related to communication and the structure of the interview. This introduction to the concepts is relevant to situate the professional on what skills will be discussed and developed. Cegala and Broz [45] point out that this is a comprehensive approach, which can reflect aspects of the interview in a natural way. Attention to the stages of the interview and the concepts and functions of communication skills can also improve the identification, coherence and particularization of interventions for training professionals.

Furthermore, inserting themes aimed at issues relevant to the target audience is essential for meaningful learning. Training should focus on the acquisition of skills and knowledge, and the promotion of affective changes, which can motivate and provoke the desire to use the new skills [36]. According to Connolly et al. [47], for behavioral change to be effective, training must keep the participant's attention and motivate them, collaborate to retain information and allow appropriate behaviors to be reproduced. These aspects can help to increase the self-efficacy and the expectation of the professionals' results.

In this perspective, the use of videos for modeling behavior and strategies such as role play, carried out in the studies included in this review, allow professionals to actively participate in the learning process. The use of simulated patients has also been encouraged, as it allows skills training in a safe environment similar to that found in the professional's reality [11]. This approach leads to an increase in the professionals’ 'perception of patients' behaviors, and identifies their reactions. This type of strategy allows the professional to develop other skills and abilities, such as respect, empathy and understanding of the patients' needs and preferences [48].

The insertion of content focused on Patient Centered Care (PCC), in the studies by Ammentorp et al. [27] and Levinson and Roter [29], suggested bringing the patient's perspective to the training center, enabling personalized attention, which considers the subjectivity of individuals and allows active participation in the care process. An important component of PCC is the communication that is established between the professional and the patient, as it allows the retrieval of relevant information about the patient's history, values, culture and preferences, which should be known and explored [49].

In view of the different content and teaching strategies, different ways to assess skills were identified. In general, the included studies showed a significant improvement in the confidence of communication skills and in the performance of health professionals. However, the performance assessed using the OSCE did not show any significant difference between groups. In contrast, Ammentorp et al. [50] identified a high agreement between the performance evaluation of communication skills and self-efficacy using OSCE in a group of medical students. Although this method of assessment is widely used in the assessment of competency-based training, Plakiotis [51] points out restrictions in measuring issues of specialized practice and it is recommended to complement it with other assessments.

In the assessment of self-efficacy, the participants submitted to the intervention had better scores. By understanding that self-efficacy is characterized by the individual's belief in his abilities to perform a task successfully, which can modify the person’s behavior [6, 13], interventions are shown as positive possibilities to improve communication skills. Even with the self-assessment barriers, improving self-efficacy can bring benefits, such as confidence, and a positive expectation can increase the likelihood of professionals using appropriate communication behaviors. Thus, thinking of instruments that allow the professional to carry out this evaluation can be an enhancer for the evaluation and success of programs [52]. Based on the moderate level of evidence verified by GRADE, professionals should be encouraged to receive the interventions recommended for improving the self-efficacy of Communication Skills, which should also be adopted for the improvement of health services.

In the perspective of knowing the effectiveness of interventions to increase self-efficacy, the exclusion of studies with other methodological designs may result in there being limited information about other training programs in communication skills, which could provide different ways of presenting the content, teaching and measures evaluation. The methodological guidance of this review aims to identify the effectiveness of training in communication skills using stronger research designs and with better assessment measures [16]. In addition, the inclusion of only one study with a long training program precludes the possibility of assessing the effect of interventions in relation to duration. The use of different assessment measures also impairs a broad comparison between the data. In this perspective, new studies with standardized measures of self-efficacy and communication skills, should be developed, in order to allow the comparability of data and determine reliable results for training effectiveness.

## Conclusion

This review presents the identification of studies that address effective strategies for training communication skills, with moderate quality of evidence by GRADE evaluation, describing the content, teaching methods and assessments worked on in the programs. This training has been proven to be relevant in improving performance and in the self-efficacy of professionals with regard to communication skills, through participant-centered strategies, which are fundamental for the improvement of care and for patient-centered attention. It is also shown that other studies should be carried out greater methodological rigor, for more reliable assessments and the construction of increasingly structured and effective programs.

In view of the findings of the review, it is suggested that the programs reserve space to discuss the basic concepts of communication and contextualize the other contents according to the scenarios in which the professionals are inserted, considering aspects of patient-centered care. They must have a sufficient workload, which allows the participant the availability of time for contextualized learning and based on experience, with strategies for modulation and the application of the skills learned, as well as for long-term monitoring strategies. The evaluations must be valid and diversified, to complement the data.

## Supplementary Information

**Additional file 1:** PROSPERO protocol.

**Additional file 2:** Chart 1. Research equations.

**Additional file 3: Figure 1**. PRISMA Flowchart.

**Additional file 4: Table 1.** Characteristics of the included studies.

**Additional file 5: Table 2.** Characteristics of communication skills training interventions.

**Additional file 6: Figure 2.** Bias risk assessment using the Cochrane collaboration tool.

**Additional file 7: Table 3.** Risk of bias in Non-RCTs (ROBINS-I).

## Data Availability

The datasets supporting the conclusions of this article are included within the article (and its additional files).

## Abbreviations

CS: Communication skills
CST: Communication skills training
RCT: Randomized controlled trials
GRADE: Grading of recommendations applicability, development and evaluation
OSCE: Objective Structured Clinical Examination
CG: Control group
IG: Intervention group
PCC: Patient-centered care
HLAQs: Health Literacy Assessment Questions
RIAS: Roter Interactional Analysis System
AFLS: Awareness, feelings, listen, solve
