# Supplementary material for: Training in communication skills for self-efficacy of health professionals: a systematic review
Source: Hum Resour Health. 2021 Mar 6;19:30. doi: 10.1186/s12960-021-00574-3 (PMC7937280; doi:10.1186/s12960-021-00574-3)
Supplement: Supplementary file 1 — Additional file 1: PROSPERO protocol. [file 12960_2021_574_MOESM1_ESM.pdf]

NHS
National Institute for Health Research

UNIVERSITY of York
Centre for Reviews and Dissemination

Systematic review

Please complete all mandatory fields below (marked with an asterisk *) and as many of the non-mandatory fields as you can then click *Submit* to submit your registration. You don't need to complete everything in one go, this record will appear in your *My PROSPERO* section of the web site and you can continue to edit it until you are ready to submit. Click *Show help* below or click on the icon
to see guidance on completing each section.
This record cannot be edited because it has been rejected

1. * Review title.

Give the working title of the review, for example the one used for obtaining funding. Ideally the title should state succinctly the interventions or exposures being reviewed and the associated health or social problems. Where appropriate, the title should use the PI(E)COS structure to contain information on the Participants, Intervention (or Exposure) and Comparison groups, the Outcomes to be measured and Study designs to be included.

Training programs in communication skills to improve self-efficacy for health personnel: systematic review and meta analysis

2. Original language title.

For reviews in languages other than English, this field should be used to enter the title in the language of the review. This will be displayed together with the English language title.

Programas de treinamento em habilidades de comunicação para melhoraria da autoeficácia dos profissionais de saúde: revisão sistemática e metanálise

3. * Anticipated or actual start date.

Give the date when the systematic review commenced, or is expected to commence.

20/05/2019

4. * Anticipated completion date.

Give the date by which the review is expected to be completed.

20/12/2019

5. * Stage of review at time of this submission.

Indicate the stage of progress of the review by ticking the relevant Started and Completed boxes. Additional information may be added in the free text box provided.
Please note: Reviews that have progressed beyond the point of completing data extraction at the time of initial registration are not eligible for inclusion in PROSPERO. Should evidence of incorrect status and/or completion date being supplied at the time of submission come to light, the content of the PROSPERO record will be removed leaving only the title and named contact details and a statement that inaccuracies in the stage of the review date had been identified.
This field should be updated when any amendments are made to a published record and on completion and publication of the review. If this field was pre-populated from the initial screening questions then you are not able to edit it until the record is published.

The review has not yet started: Yes

Review stage	Started	Completed
Preliminary searches	No	No
Piloting of the study selection process	No	No
Formal screening of search results against eligibility criteria	No	No
Data extraction	No	No
Risk of bias (quality) assessment	No	No
Data analysis	No	No

Provide any other relevant information about the stage of the review here (e.g. Funded proposal, protocol not yet finalised).

6. * Named contact.

The named contact acts as the guarantor for the accuracy of the information presented in the register record.

Ádala Nayana de Sousa Mata

Email salutation (e.g. "Dr Smith" or "Joanne") for correspondence:

Ms Ádala Mata

7. * Named contact email.

Give the electronic mail address of the named contact.

adalamata@gmail.com

8. Named contact address

Give the full postal address for the named contact.

9. Named contact phone number.

Give the telephone number for the named contact, including international dialling code.

+55 (84) 99991-2065

10. * Organisational affiliation of the review.

Full title of the organisational affiliations for this review and website address if available. This field may be completed as 'None' if the review is not affiliated to any organisation.

Universidade Federal do Rio Grande do Norte (UFRN)

Organisation web address:

11. * Review team members and their organisational affiliations.

Give the personal details and the organisational affiliations of each member of the review team. Affiliation refers to groups or organisations to which review team members belong. **NOTE: email and country are now mandatory fields for each person.**

Professor Ádala Nayana de Sousa Mata. Universidade Federal do Rio Grande do Norte (UFRN)
Professor Liliane Pereira Braga. Universidade Federal do Rio Grande do Norte (UFRN)
Ms Kesley Pablo Morais de Azevedo. Universidade Federal do Rio Grande do Norte (UFRN)
Isac Davison. Universidade Federal do Rio Grande do Norte (UFRN)
Isaac Newton. Universidade Federal do Rio Grande do Norte (UFRN)
Miss Jessica S. Universidade Federal do Rio Grande do Norte (UFRN)
Ismael Martínez Nicolas. Universidad Católica San Antonio de Murcia (UCAM), Espanha
Grasiela Piuvezam. Universidade Federal do Rio Grande do Norte (UFRN), Brazil

12. * Funding sources/sponsors.

Give details of the individuals, organizations, groups or other legal entities who take responsibility for initiating, managing, sponsoring and/or financing the review. Include any unique identification numbers assigned to the review by the individuals or bodies listed.

Not applicable

Grant number(s)

13. * Conflicts of interest.

List any conditions that could lead to actual or perceived undue influence on judgements concerning the main topic investigated in the review.

None

14. Collaborators.

Give the name and affiliation of any individuals or organisations who are working on the review but who are not listed as review team members. **NOTE: email and country are now mandatory fields for each person.**

15. * Review question.

State the question(s) to be addressed by the review, clearly and precisely. Review questions may be specific or broad. It may be appropriate to break very broad questions down into a series of related more specific questions. Questions may be framed or refined using PI(E)COS where relevant.

RQ1: What training programs in communication skills are effective in promoting the self-efficacy in communication of health professionals?

RQ2: What type of structure and content, evaluation methods and results are used in effective communication training?

P – Health professionals

I – Communication skills training

C – Heath professionals submitted to communication skills training (with and without a control group)

O – Self efficacy or improvements of professionals' communication skills

S - Experimental studies

16. * Searches.

State the sources that will be searched. Give the search dates, and any restrictions (e.g. language or publication period). Do NOT enter the full search strategy (it may be provided as a link or attachment.)

A comprehensive search of the following databases was carried out: PubMed/MEDLINE, Scopus, Web of Science, EMBASE, ScienceDirect, CINAHL, PsycINFO and the Cochrane Central Register of Controlled Trials (CENTRAL). A combination of free text search terms and Medical Subject Title (MeSH), text words and keywords.

The search strategy can be summarised as a key word search of :

word group 1: health personnel OR health care providers OR health care workers

AND word group 2: communication OR empathy OR clinical competence OR clinical skills OR professional patient relations OR patient-centered care

AND word group 3: education OR training program OR workshop

AND word group 4: self efficacy

The search terms used for the formations of the search equations will be combined with specific filters of each database. There will be no limitation of time and languages in the searches to be performed.

17. URL to search strategy.

Give a link to a published pdf/word document detailing either the search strategy or an example of a search strategy for a specific database if available (including the keywords that will be used in the search strategies), or upload your search strategy.Do NOT provide links to your search results.

https://www.crd.york.ac.uk/PROSPEROFILES/129384_STRATEGY_20190322.pdf

Alternatively, upload your search strategy to CRD in pdf format. Please note that by doing so you are consenting to the file being made publicly accessible.

Yes I give permission for this file to be made publicly available

18. * Condition or domain being studied.

Give a short description of the disease, condition or healthcare domain being studied. This could include health and wellbeing outcomes.

Studies on communication skills in different health contexts have been carried out, with the concern of improving the relationship between professionals and their patients. However, most of the studies have as results the evaluation of patient satisfaction, improvement of health parameters or improvement in the professional-patient relationship after training. In this way, we intend to evaluate the results of the communication skills training, in the perspective of the change of the professional competences through the

evaluation of the self-efficacy.

19. * Participants/population.

Give summary criteria for the participants or populations being studied by the review. The preferred format includes details of both inclusion and exclusion criteria.

Will include studies:

1. focused on communication skills training
2. performed with health professionals
3. report a change in professional self-efficacy or other attitudinal and behavioral changes

The Exclusion Criteria:

1. Studies conducted with undergraduate or graduate students
2. Construction of questionnaires to verify communication skills
3. systematic reviews
4. interventions performed by mindfulness programs
5. Interventions using psychotherapy

20. * Intervention(s), exposure(s).

Give full and clear descriptions or definitions of the nature of the interventions or the exposures to be reviewed.

Usually, the training programs use different duration periods and strategies, such as reading texts, simulation, role play, etc. In this study, structured interventions to improve the communication skills of health professionals should be considered, with definition of content, time and evaluation of the results, associated to improvement of professional performance (self-efficacy).

21. * Comparator(s)/control.

Where relevant, give details of the alternatives against which the main subject/topic of the review will be compared (e.g. another intervention or a non-exposed control group). The preferred format includes details of both inclusion and exclusion criteria.

Studies with and without a control group will be included in this review. The results of different communication skills training programs will be compared in this review.

22. * Types of study to be included.

Give details of the types of study (study designs) eligible for inclusion in the review. If there are no restrictions on the types of study design eligible for inclusion, or certain study types are excluded, this should be stated. The preferred format includes details of both inclusion and exclusion criteria.

Clinical trials (Randomised, non-randomised), before and after studies, observational estudies (prospective

and retrospective) and qualitative studies will be eligible for inclusion.

23. Context.

Give summary details of the setting and other relevant characteristics which help define the inclusion or exclusion criteria.

24. * Main outcome(s).

Give the pre-specified main (most important) outcomes of the review, including details of how the outcome is defined and measured and when these measurement are made, if these are part of the review inclusion criteria.

The results may include:

1. Improvement in self-efficacy in the communication skills of professionals;
2. Improvement in communication skills of health professionals;
3. Improvement in behavior or attitude in health professionals.

* Measures of effect

Please specify the effect measure(s) for you main outcome(s) e.g. relative risks, odds ratios, risk difference, and/or 'number needed to treat.

25. * Additional outcome(s).

List the pre-specified additional outcomes of the review, with a similar level of detail to that required for main outcomes. Where there are no additional outcomes please state ‘None’ or ‘Not applicable’ as appropriate to the review

The impact on patient satisfaction outcome due to communication training.

* Measures of effect

Please specify the effect measure(s) for you additional outcome(s) e.g. relative risks, odds ratios, risk difference, and/or 'number needed to treat.

26. * Data extraction (selection and coding).

Describe how studies will be selected for inclusion. State what data will be extracted or obtained. State how this will be done and recorded.

Two authors will independently screen the search results using titles and abstracts, and full text. This step will be carried out by your researchers: ANSM, LPB, ID, IN and IMN. Duplicates and reviews will be removed from the database. Reviewers will then go through the full text to determine whether they meet the inclusion criteria. References cited in articles will be further reviewed to locate any additional relevant articles not retrieved within the primary search. Discrepancies will be resolved by a third reviewer, GP. The selection of the studies summarized in a PRISMA flow diagram.

Data collected will include relevant information on population characteristics, study setting, type of communication education strategy used (brief description), study methods (length of intervention, length of follow-up, data collection points, inclusion criteria and method of randomization, if applicable), supporting evidence for educational strategy, effectiveness measurements, description of each of the interventions and of each of the comparators, outcomes of significance to the review question.

27. * Risk of bias (quality) assessment.

Describe the method of assessing risk of bias or quality assessment. State which characteristics of the studies will be assessed and any formal risk of bias tools that will be used.

Two independent reviewers will use the Cochrane risk of bias tool to assess random sequence generation, allocation concealment, blinding of participants, clinicians and outcome assessment. In addition, incomplete outcome data, selective reporting, funding, and potential for conflicts of interest associated with the individual trials will also be considered. The risk of bias will be rated using predetermined criteria as follows: low, high or unclear. We will attempt to obtain any missing data by contacting the first or corresponding authors or coauthors of an article via phone, email or post. If we fail to receive any necessary information, the data will be excluded from our analysis and will be addressed in the Discussion section. The heterogeneity between trial results will be evaluated using a standard X^2 test with a significance level 0, 05. To assess heterogeneity, we plan to compute the I^2statistic, which is a quantitative measure of inconsistency across studies. A value of 0% indicates no observed heterogeneity, whereas I^2 values of 50% indicate a substantial level of heterogeneity. If possible, funnel plots will be used to assess the presence of potential reporting biases. A linear regression approach will be used to evaluate funnel plot asymmetry.

28. * Strategy for data synthesis.

Provide details of the planned synthesis including a rationale for the methods selected. This **must not be generic text** but should be **specific to your review** and describe how the proposed analysis will be applied to your data.

This will be carried out using the Rev Man Analyses statistical package in Review Manager V.5.1. For dichotomous outcomes, we will derive the OR and 95% CI for each study. The heterogeneity between the trial results will be evaluated using a standard I^2 test with a significance level of p 0.1, and the I^2 statistic, which is a quantitative measure of inconsistency across studies, with a value of 0% indicating no observed heterogeneity, and values of 50% indicating substantial levels are present. If there is heterogeneity (I^2 75%), a random-effects model will be used to combine the trials to calculate the relative risk (RR) and 95% CI, using the DerSimonian-Laird algorithm in meta for package, a meta-analysis package for R. Other study characteristics and results will be summarised narratively if a meta-analysis cannot be performed for all or some of the included studies. If possible, funnel plots will also be used to assess the presence of potential reporting biases, and a linear regression approach will be used to evaluate funnel plot asymmetry.

29. * Analysis of subgroups or subsets.

State any planned investigation of ‘subgroups’. Be clear and specific about which type of study or participant will be included in each group or covariate investigated. State the planned analytic approach.

The analysis of the subgroups was performed considering the types of intervention, and the results presented regarding the effectiveness of professionals in communication skills.

30. * Type and method of review.

Select the type of review and the review method from the lists below. Select the health area(s) of interest for your review.

Type of review

Cost effectiveness
No

Diagnostic
No

Epidemiologic
No

Individual patient data (IPD) meta-analysis
No

Intervention
Yes

Meta-analysis
Yes

Methodology
No

Narrative synthesis
No

Network meta-analysis
No

Pre-clinical
No

Prevention
No

Prognostic
No

Prospective meta-analysis (PMA)
No

Review of reviews
No

Service delivery
No

Synthesis of qualitative studies
No

Systematic review
Yes

Other
No

Health area of the review

Alcohol/substance misuse/abuse
No

Blood and immune system
No

Cancer
No

Cardiovascular

No

Care of the elderly
No

Child health
No

Complementary therapies
No

Crime and justice
No

Dental
No

Digestive system
No

Ear, nose and throat
No

Education
Yes

Endocrine and metabolic disorders
No

Eye disorders
No

General interest
No

Genetics
No

Health inequalities/health equity
No

Infections and infestations
No

International development
No

Mental health and behavioural conditions
No

Musculoskeletal
No

Neurological
No

Nursing
No

Obstetrics and gynaecology
No

Oral health
No

Palliative care
No

Perioperative care
No

Physiotherapy
No

Pregnancy and childbirth
No

Public health (including social determinants of health)
Yes

Rehabilitation
No

Respiratory disorders
No

Service delivery
No

Skin disorders
No

Social care
Yes

Surgery
No

Tropical Medicine
No

Urological
No

Wounds, injuries and accidents
No

Violence and abuse
No

31. Language.

Select each language individually to add it to the list below, use the bin icon to remove any added in error.
English
Portuguese-Brazil

There is an English language summary.

32. * Country.

Select the country in which the review is being carried out from the drop down list. For multi-national collaborations select all the countries involved.

Brazil

33. Other registration details.

Give the name of any organisation where the systematic review title or protocol is registered (such as with The Campbell Collaboration, or The Joanna Briggs Institute) together with any unique identification number assigned. (N.B. Registration details for Cochrane protocols will be automatically entered). If extracted data will be stored and made available through a repository such as the Systematic Review Data Repository (SRDR), details and a link should be included here. If none, leave blank.

34. Reference and/or URL for published protocol.

Give the citation and link for the published protocol, if there is one

Give the link to the published protocol.

Alternatively, upload your published protocol to CRD in pdf format. Please note that by doing so you are consenting to the file being made publicly accessible.

No I do not make this file publicly available until the review is complete

Please note that the information required in the PROSPERO registration form must be completed in full even if access to a protocol is given.

35. Dissemination plans.

Give brief details of plans for communicating essential messages from the review to the appropriate audiences.

We plan to publish the paper in scientifc journals.

Do you intend to publish the review on completion?

Yes

36. Keywords.

Give words or phrases that best describe the review. Separate keywords with a semicolon or new line. Keywords will help users find the review in the Register (the words do not appear in the public record but are included in searches). Be as specific and precise as possible. Avoid acronyms and abbreviations unless these are in wide use.

37. Details of any existing review of the same topic by the same authors.

Give details of earlier versions of the systematic review if an update of an existing review is being registered, including full bibliographic reference if possible.

38. * Current review status.

Review status should be updated when the review is completed and when it is published. For newregistrations the review must be Ongoing.
Please provide anticipated publication date

Review_Ongoing

39. Any additional information.

Provide any other information the review team feel is relevant to the registration of the review.

40. Details of final report/publication(s).

This field should be left empty until details of the completed review are available.

Give the link to the published review.
