# Supplementary material for: Training in communication skills for self-efficacy of health professionals: a systematic review
Source: Hum Resour Health. 2021 Mar 6;19:30. doi: 10.1186/s12960-021-00574-3 (PMC7937280; doi:10.1186/s12960-021-00574-3)
Supplement: Supplementary file 2 — Additional file 2: Chart 1. Research equations. [file 12960_2021_574_MOESM2_ESM.docx]

| **DATABASE** | **RESEARCH EQUATIONS** |
| --- | --- |
| PUBMED/MEDLINE | ("health personnel" OR "health care providers" OR "health care workers") AND ("communicat*" OR "communication" OR "empathy" OR "clinical skills" OR "professional patient relations" OR "patient-centered care") AND ("education" OR "training program" OR "workshop") AND ("self efficacy") |
| CINHAL | ("health personnel" OR "health care providers" OR "health care workers") AND ("communicat*" OR "communication" OR "empathy" OR "clinical skills" OR "professional patient relations" OR "patient-centered care") AND ("education" OR "training program" OR "workshop") AND ("self efficacy") |
| PSYCINFO | ("health personnel" OR "health care providers" OR "health care workers") AND ("communicat*" OR "communication" OR "empathy" OR "clinical skills" OR "professional patient relations" OR "patient-centered care") AND ("education" OR "training program" OR "workshop") AND ("self efficacy") |
| SCIENCE DIRECT | (“health personnel”) AND (“communication” OR “empathy” OR “professional patient relations” OR “patient-centered care”) AND (“training program” OR “workshop”) AND (“self efficacy”) |
| SCOPUS | (“health personnel” OR “health care providers” OR “health care workers”) AND (“communicat*” OR “empathy” OR “professional patient relations” OR “patient-centered care”) AND (“training program” OR “workshop”) AND (“self efficacy”) |
| WEB OF SCIENCE | TS=(("health personnel" OR "health care providers" OR "health care workers") AND ("communicat*" OR "communication" OR "empathy" OR "clinical skills" OR "professional patient relations" OR "patient-centered care") AND ("education" OR "training program" OR "workshop") AND ("self efficacy")) |
| EMBASE | (("health personnel" OR "health care providers" OR "health care workers") AND ("communicat*" OR "empathy" OR "professional patient relations" OR "patient-centered care") AND ("training program" OR "workshop") AND ("self efficacy")) |
| COCHRANE CENTRAL | ("health personnel" OR "health care providers" OR "health care workers") AND ("communicat*" OR "communication" OR "empathy" OR "clinical skills" OR "professional patient relations" OR "patient-centered care") AND ("education" OR "training program" OR "workshop") AND ("self efficacy") |
