# Supplementary figures and images for: Training in communication skills for self-efficacy of health professionals: a systematic review
Source: Hum Resour Health. 2021 Mar 6;19:30. doi: 10.1186/s12960-021-00574-3 (PMC7937280; doi:10.1186/s12960-021-00574-3)

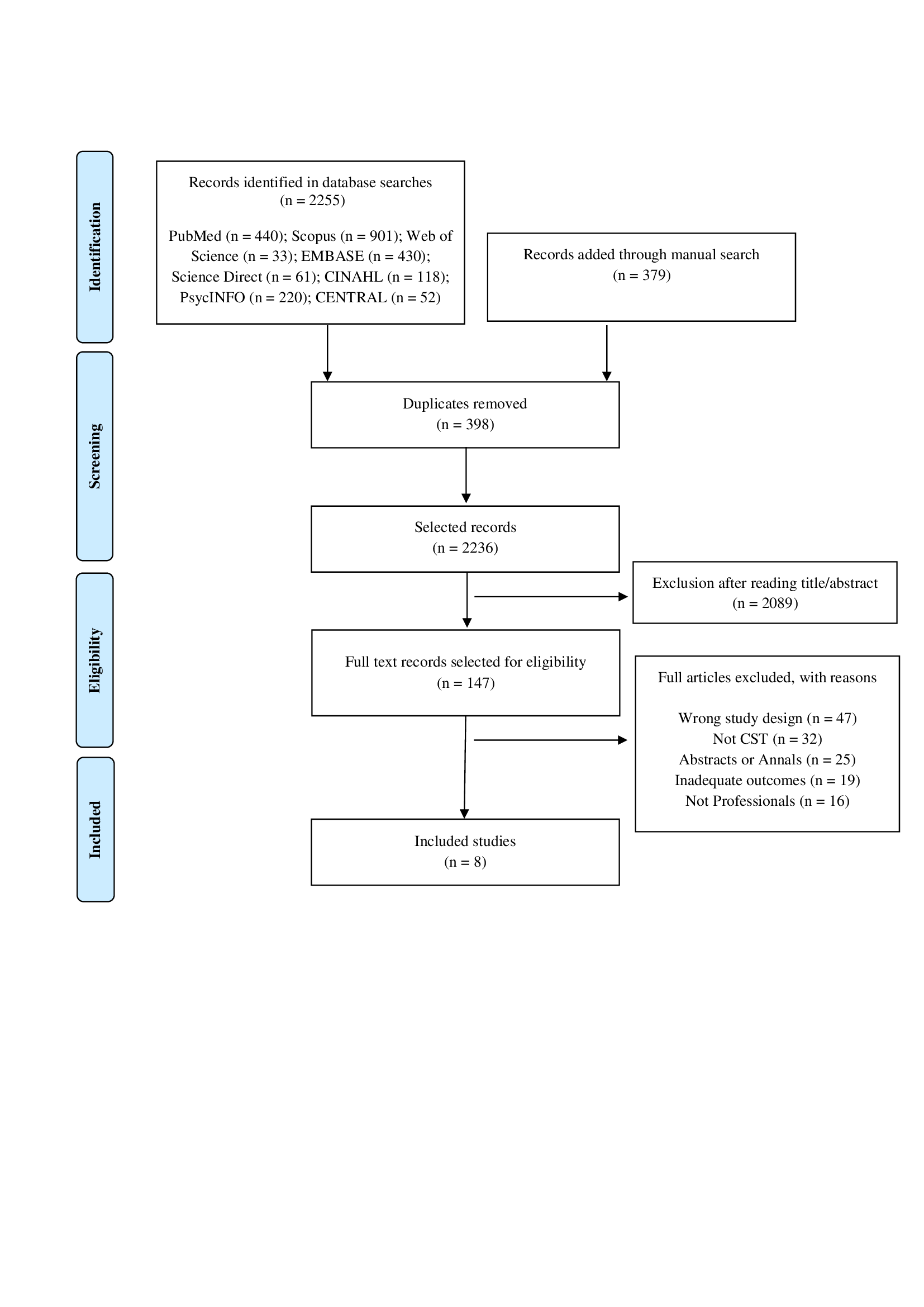

Supplement: Supplementary file 3 — Additional file 3: Figure 1. PRISMA Flowchart. [file 12960_2021_574_MOESM3_ESM.tiff]

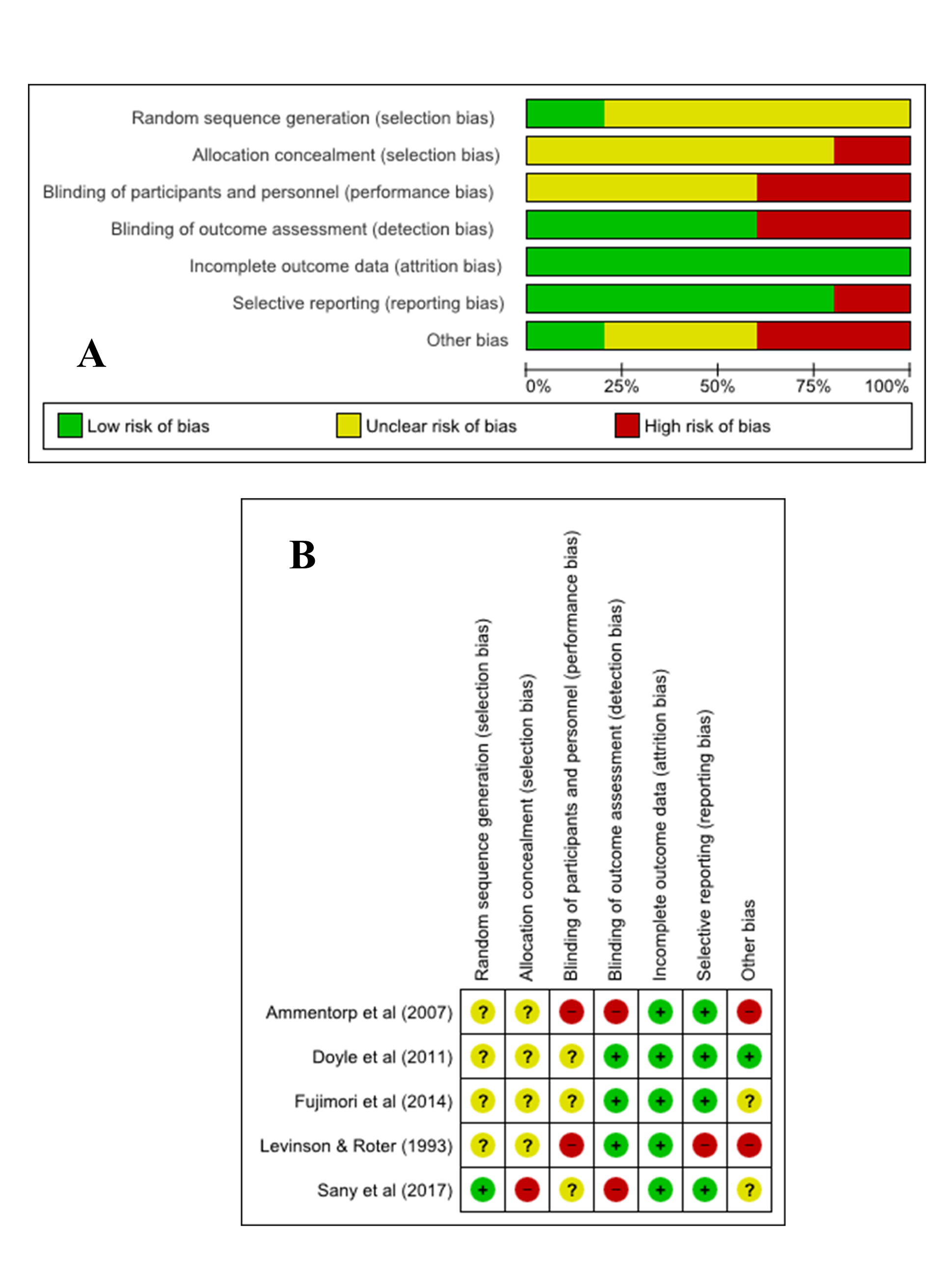

Supplement: Supplementary file 6 — Additional file 6: Figure 2. Bias risk assessment using the Cochrane collaboration tool. [file 12960_2021_574_MOESM6_ESM.tif]
