# Supplementary material for: Training in communication skills for self-efficacy of health professionals: a systematic review
Source: Hum Resour Health. 2021 Mar 6;19:30. doi: 10.1186/s12960-021-00574-3 (PMC7937280; doi:10.1186/s12960-021-00574-3)
Supplement: Supplementary file 4 — Additional file 4: Table 1. Characteristics of the included studies. [file 12960_2021_574_MOESM4_ESM.docx]

**Table 1.** **Characteristics of the included studies.**

| **Author** | **Year** | **Country** | **Desing** | **Clinical Context** | **Population** | **n** | **Age (Average)** | **Gender Women %** |
| --- | --- | --- | --- | --- | --- | --- | --- | --- |
| Sany et al. | 2017 | Iran | RCT | Primary care, hypertension | Doctors, nutritionists, nurses or medical assistants | 35 | 37.08 | 64 |
| Fujimori et al. | 2014 | Japan | RCT | Hospital, Cancer | Doctors | 30 | 38 | 86.7 |
| Doyle et al. | 2011 | USA | RCT | Primary care | Nurses | 33 | NR | NR |
| Ammentorp et al. | 2007 | Denmark | RCT | Outpatient,  pediatric | Doctors and nurses | 29 | NR | 72.4 |
| Liu et al. | 2007 | China | Quasi-experimental | Hospital, cancer | Nurses | 117 | 30.38 | 100 |
| van Dulmen and Holl | 2000 | Netherlands | Quasi-experimental | Hospital, Pediatric | Doctors | 21 | 45 | 42,9 |
| Roter et al. | 1998 | USA | Quasi-experimental | Outpatient | Doctors | 15 | NR | 13,3 |
| Levinson and Roter | 1993 | USA | RCT | Primary care | Doctors | 30 | NR | NR |

RCT – Randomized Controlled Trials; USA – United States of America; NR – Not Reported
