# Supplementary material for: Training in communication skills for self-efficacy of health professionals: a systematic review
Source: Hum Resour Health. 2021 Mar 6;19:30. doi: 10.1186/s12960-021-00574-3 (PMC7937280; doi:10.1186/s12960-021-00574-3)
Supplement: Supplementary file 5 — Additional file 5: Table 2. Characteristics of communication skills training interventions. [file 12960_2021_574_MOESM5_ESM.docx]

**Table 2. Characteristics of communication skills training interventions.**

| **Author (year)** | **Training duration** | **Follow-up** | **Content** | **Teaching strategies** | **Evaluated behaviors**  **– Assessment tools** | **Main results** |
| --- | --- | --- | --- | --- | --- | --- |
| Sany et al. (2017) | 1 day | - | *First Workshop*  Spoken Communication and Written Communication  *Second Workshop*  Ability to collaborate, to improve self-care behavior and adherence to medications and Primary care support to develop medical counseling skills and patient self-efficacy. | *First Workshop*  Spoken communication: Video, Follow-up instruction form, MedCard, review poster and Written communication: Medical information release, Results letter, Useful posters and Educational materials  *Second Workshop*  Collaboration skills: Video, PowerPoint, poster, Pills graphic; Didactic presentations and Primary care support: Medical progress notes, role play and didactic presentations. | Communication Skills – Health Literacy Assessment Questions (HLAQs). | Significant evidence was found in the change in communication skills of the professional from baseline to follow-up, as well as the intervention group achieved significant improvement compared to the control group. |
| Fujimori et al. (2014) | 2 days | - | Conceptual communication skills model: SHARE.  Module: Introduction, communication skills model, evidence of patient preferences in relation to communication; 2. Trigger video: Communicate bad news; 3. Role playing: Communicating bad news using scenarios; 4. Peer discussion: role play, final summary. | Orientation; breaking of ice; lecture; video trigger; role play with peer discussion; summarization. | Performance in communication skills - Evaluation of simulated queries by SHARE items;  Confidence in communicating with patients - 32 SHARE items and questionnaire Baile et al. | In performance, assessed by the SHARE categories, significant differences were revealed between the groups in three of the four factors and seven of the 27 categories. There were differences in the mean scores of confidence in SHARE communication between groups and communication of bad news between groups. |
| Doyle et al. (2011) | 2 sessions of 3h30min  Interval 1 or 2 weeks between sessions | - | Model AFLS (awareness, feelings, listen, solve).  During the first session, modules on the overview of the AFLS process, facilitating listening, non-verbal communication and assertive communication. Between sessions, participants had reading and observation tasks to complete. In the second session, modules on conflict resolution were addressed. | Pedagogy of experimental learning, student-centered.  Each session included mini lectures to introduce information and describe skills. The skills were modeled by videos or role play. Participants worked in dyads to reflect on personal experience, brainstorm and write their own scenarios. Small group role play was used for practice and feedback. Each participant received a set of exercises, reading material and a pocket card listing the basic steps of the AFLS process. | Self-efficacy - 2 scales: the confidence scale Parle et al., [26] and the extent of difficulty scale Arranz et al.;  Performance - Evaluates the OSCE through 18 items. | Nurses who participated in the course ranked significantly higher in their self-efficacy compared to those who did not participate in the course. Participants improved their performance scores from pre to post-OSCE, but the analysis of OSCE post-test scores showed no significant difference between the intervention and control groups. |
| Ammentorp et al. (2007) | 2 sessions of 3 and 2 days, and a period of 4 weeks separates the sessions | 3 and 6 months | Based on the method described by Maquire et al.  The course comprised three basic elements: 1. Structure, a rigid consultation structure, to ensure that all relevant aspects are considered, in order to guarantee an overview and transparency. 2. Communication techniques, to learn to listen, how to help the patient to formulate problems and how to ask the right questions, etc. 3. Patient-centered, raising and understanding the patient's concerns and needs, with the patient achieving a mutual understanding of the problem and the treatment. | Initially, the participants identified the communication tasks that most wanted help. A video demonstrating an evaluation model is shown and discussed about communication behaviors. Participants, divided into smaller groups, to practice specific communication tasks through role play and feedback. In the four-week period that separated the two parts of the course, participants rehearsed and videotaped one of their own consultations that were used to give feedback to participants during the last 2 days of the course. | Self-efficacy - Questionnaire developed by Parle et al. | Before the course, the difference between the control group and the intervention group was small and not statistically significant. After the intervention, the general self-efficacy was statistically significantly higher in the intervention group just after the course (T2) and 6 months after the course (T4). |
| Liu et al. (2007) | 3-day (21-hour) course over a 3-week period | **-** | Intensive learning in a big group: The ICSTP was developed as a learner-centered program, incorporating cognitive, affective, and behavioral components, as well as managerial support, based on the Integrated Communication Skills Training Model (Parle et al., 1997).  Professional identifies, through group discussion, the communication tasks for which they most need help when talking to cancer patients, family members or colleagues. | Intensive learning in a big group: lecture, a video demonstration, question and discussion, and a video review.  After having finished the intensive learning session, the following section was to empower the head nurses, together with their clinical instructors, to organize their clinical unit-based practice in small groups. The methods of managerial support include giving nurses positive feedback, establishing a peer-supportive atmosphere, implementing teaching rounds, building up role models, and conducting roleplaying within small groups in their workplace. A handbook of training materials was distributed to each nurse participant in the training group. | Nurses’ Basic Communication Skills Scale (NBCSS); Nurses’ Self-Efficacy Ratings in Oncology Specified Communication Tasks scale (NSROSCT); Communication Outcomes Questionnaire (COQ); Nurses’ Self-Perceived Support Scale (NSSS). | The findings show that the nurses in the training group significantly increased in their mean scores for all the four dependent variables (Basic communication Skills; Self-efficacy; Outcome expectancy; Perceived support) in T3, compared with the mean scores in T1. In contrast, no significant difference was found for the mean scores of nurses between T3 and T1 in the control group over the same period |
| van Dulmen and Holl (2000) | 5 days | **-** | Patient-centred interviewing skills and handle parents' and children's instrumental and affective needs, by providing information and advice and showing support and understanding | 1) paediatrician education on the significance of communicating affect, of attending to psychosocial issues, and of giving the patient and the parent room to talk; 2) theoretical and practical homework and application of what was learned between sessions; 3) role-playing exercises, for becoming comfortable with alternative communication skills; 4) trainers' and colleagues' feedback of videotaped and role-play interaction style and 5) discussion of paediatricians' own experiences. | All participants were asked to videotape a series of consecutive outpatient visits.  Paediatricians' verbal and nonverbal communication was measured by four independent raters directly from the video recordings using the CAMERA computer system. | At post-measurement, trained paediatricians appeared to express more agreements, to provide more medical information, and to ask more psychosocial questions.Significant differences were found on asking psychosocial questions and on patient-directed gaze between the post-measurements of the control and the experimental group |
| Roter et al. (1998) | 8 hours, 2 days | **-** | Overview of the literature and rationale for the program describing basic research regarding the links between interpersonal communication and patient and doctor outcomes; presentation of the results of the baseline study conducted prior to training;  practice of specific communication skills - in three dimensions: informativeness, emotional responsiveness, and partnership building. | Role play  Detailed and annotated manual defining the target skills, transcripts of sample medical visits, an overview of patient and doctor questionnaire results, and selected readings and bibliography | Roter Interaction Analysis System (RIAS). | Trained doctors used significantly more facilitations in their visits and more open-ended questions. |
| Levinson and Roter (1993) | 4 ½ hour workshop | - | Conducted by Miles Program for Physician-Patient Communication.  Fundamental skills in medical interviewing, patient participation, empathic communication, patient education and patient involvement in health care discussions. | Didactic presentations and case-based discussions, focusing on fundamental skills in the medical interview. | Communication skills - Analysis of recorded and evaluated queries using the Roter Interactional Analysis System (RIAS). | There was no evidence of an effect of short-term training in any of the analyses. |
