# Supplementary material for: Training in communication skills for self-efficacy of health professionals: a systematic review
Source: Hum Resour Health. 2021 Mar 6;19:30. doi: 10.1186/s12960-021-00574-3 (PMC7937280; doi:10.1186/s12960-021-00574-3)
Supplement: Supplementary file 7 — Additional file 7: Table 3. Risk of bias in Non-RCTs (ROBINS-I). [file 12960_2021_574_MOESM7_ESM.docx]

**Table 3. Risk of bias in Non-RCTs (ROBINS-I).**

|  | **Confounding** | **Selection of participants** | **Classification of interventions** | **Departures from intended interventions** | **Missing data** | **Measurement of outcomes** | **Selection of reported results** | **Overall judgment** |
| --- | --- | --- | --- | --- | --- | --- | --- | --- |
| Liu et al. (2007) | Low | Low | Low | Low | Low | Moderate | Low | Moderate |
| van Dulmen & Hall (2000) | Low | Low | Low | Low | Low | Moderate | Low | Moderate |
| Roter et al. (1998) | Low | Low | Moderate | Low | Moderate | Moderate | Low | Moderate |
